# Urbanized lands degrade surrounding grasslands by deteriorating the interactions between plants and soil microbiome

**DOI:** 10.3389/fmicb.2024.1505916

**Published:** 2025-01-06

**Authors:** Mengchao Fang, Guang Lu, Shuping Zhang, Wei Liang

**Affiliations:** ^1^College of Life and Environment Science, Minzu University of China, Beijing, China; ^2^Ministry of Education Key Laboratory for Ecology of Tropical Islands, College of Life Sciences, Hainan Normal University, Haikou, China

**Keywords:** urbanization, grassland ecosystem, plant community, soil microbial community, degradation

## Abstract

To mitigate overgrazing on grasslands, towns were constructed in some pastoral regions of China to relocate pastoralists. Nevertheless, whether and how the urbanized lands impact the surrounding grassland ecosystem remains unclear. We assessed the impacts of urbanized lands on the plant and soil interactions within the surrounding grasslands in order to ensure an eco-sustainable pastoralist relocation. The town with 1 km radius was selected as urbanization sample and a grassland with 1 km radius was selected as nature grassland sample. Plants and soil were investigated in nature grassland (NG), and areas 1 km (T-1 km), 2 km (T-2 km), and 3 km (T-3 km) from the center of the town. In T-1 km and T-2 km, compared to the NG, plant diversity, the abundance of dominant plant species, the abundance of soil wood saprotroph fungi, soil water content (SWC), and total organic carbon (TOC) decreased, while soil plant pathogen fungi, soil pH, and total phosphatase (TP) increased. Conversely, no such changes were observed in T-3 km. The results of Mantel test and Partial least squares path model suggest that the decrease in soil TOC and SWC, along with the increase in pH and TP in T-1 km and T-2 km, lead to a decline in wood saprotroph fungi and an increase in plant pathogen fungi, ultimately resulting in reductions in plant diversity and the abundance of dominant plant species. These results indicate that towns in pastoral areas can lead to surrounding grassland degradation by deteriorating the plant–soil interactions.

## 1 Introduction

Grassland is an essential terrestrial ecosystem type that provides natural resources for pastoralists (Sun et al., 2022). Biodiversity is the cornerstone of maintaining the stability of the grassland ecosystem (Halliday et al., 2020; Sun et al., 2022), which is intimately tied to the livelihoods of pastoralists. In turn, moderate grazing can expedite the material cycle within the grassland, thereby sustaining its biodiversity (Davinic et al., 2013; Xun et al., 2018). Therefore, achieving a balance between grassland conservation and the sustainable livelihoods of pastoralist is an important ecological and social issue. Due to population growth, overgrazing has led to significant grassland degradation across China over the past three decades (Niu et al., 2019; Feng et al., 2023; Wang et al., 2023). To mitigate overgrazing and restore degraded grasslands, towns were established in pastoral areas, aimed at encouraging pastoralists to relocate and transition into industries and services (Song et al., 2015; Yeh and Makely, 2019; Gongbuzeren et al., 2021). Nevertheless, urbanization has been recognized as the primary anthropogenic force that negatively impacts the biodiversity of natural ecosystems by deteriorating soil and vegetation (Simkin et al., 2022; Yang et al., 2022a; Wang et al., 2018; Ren et al., 2022; Ruas et al., 2022; Toledo-Garibaldi et al., 2023; Kurylo et al., 2024). While “pastoralist relocation to towns” mitigated grazing pressure, the detrimental consequences of urbanization on the grassland ecosystem have been overlooked. To ensure an eco-sustainable pastoralist relocation, it is imperative to evaluate the ecological processes and consequences that urbanized lands impose on biodiversity of the grassland ecosystem.

Urbanization involves the expansion of impermeable areas onto natural land, disrupting the natural water cycle within urban regions and their vicinities (Hu et al., 2018; Wu et al., 2024). Furthermore, it brings about environmental alterations such as the heat island effect and air pollution (Yang et al., 2022a; Yao et al., 2023). Consequently, an ecotone emerges at the interface between urban and natural landscapes, characterized by distinct conditions in terms of water availability, temperature, and atmospheric composition compared to those observed in natural ecosystems (Goodarzi et al., 2020; Alue et al., 2022; Tatsumi et al., 2023). In the edge areas induced by anthropogenic disturbances, the deterioration of temperature, moisture, light, and soil physicochemical properties can adversely impact the survival and interactions of plants and soil microorganisms, leading to a “negative edge effect” (van der Heijden et al., 2008; Bardgett and van der Putten, 2014; Schmidt et al., 2017; Koelemeijer et al., 2023). In this process, vegetation degradation caused by anthropogenic disturbances in the affected areas can lead to a decrease in litter and alterations in plant root exudates. These changes subsequently impact the composition and function of soil microbial communities by diminishing nutrient sources and reducing the overall suitability of the soil environment (Cline and Zak, 2016; van der Heijden et al., 2016; Delgado-Baquerizo et al., 2018). Changes in soil microbial communities can alter organic matter decomposition and nutrient cycling within the soil, thereby influencing plant growth and development, ultimately leading to a reduction in plant diversity (Bardgett and van der Putten, 2014; Smith et al., 2015; Wang et al., 2022b). This cascade can propagate, causing the degradation of adjacent areas by influencing the soil properties of neighboring areas (Fang et al., 2024). Numerous studies show that in edge zones induced by anthropogenic disturbances, the harmonious interactions between soil microorganisms and plant communities are disrupted, resulting in a marked decline in soil nutrient cycling rate (Pennanen, 2001; Malmivaara-Lämsä et al., 2008; Fang et al., 2024). Recent investigations at the urban-forest interface have revealed that soil respiration rates diminish due to elevated temperatures and arid conditions (Carey et al., 2016; Garvey et al., 2022; Garvey et al., 2023), while the mutually beneficial relationship between trees and their fungal root symbionts deteriorates (Tatsumi et al., 2023). These findings in urban-forest edges imply that urbanized lands contribute to the degradation of adjacent natural ecosystems by impairing the intricate interplay between plants and soil.

The urbanization induced negative edge effects on the plant–soil interaction may be particularly pronounced in fragile grassland ecosystems due to their inherently poor soil quality and low precipitation levels (Gao B. et al., 2023; Liu et al., 2024). However, the urbanization induced negative edge effects remain unclear. Grasslands are distributed in arid or semi-arid regions, where precipitation is low and soil moisture evaporates rapidly (Fan et al., 2024). The vegetation of grasslands comprises herbaceous plants with less developed roots, and the litter from these plants decomposes rapidly at the soil surface, resulting in insufficient nutrient accumulation in the soil (Li H. et al., 2024). Consequently, in fragile grassland ecosystems, soil microorganisms are highly sensitive to variations in water and soil nutrients, making the interactions between plants and soil microorganisms more susceptible to disruption (Han et al., 2024).

The urbanization disturbances may exacerbate the interactions between grassland plants and soil microorganisms, ultimately resulting in a decline in plant diversity and vegetation degradation. Furthermore, studies of forests have revealed that the negative edge effects can be observed within a specific range surrounding urbanized lands (Harper et al., 2005; Ewers and Didham, 2006; Smith and Smith, 2010; Vallet et al., 2010; Veselkin et al., 2018). Hence, it is imperative to investigate the extent to which the negative edge effect can be detected in surrounding grasslands. This knowledge is crucial for planning the size and density of the towns, with the aim of mitigating the detrimental effects of urbanization on the grassland ecosystem.

The Hulunbuir grassland, located in the Inner Mongolia of China, is a typical temperate meadow grassland and an important pastoral region in China (Sun et al., 2016). Since the 1980s, the local government has established towns of varying sizes within the pastoral areas to protect the grassland from overgrazing. In this study, we take the Hulunbuir grassland as a case study to investigate whether and to what extent urbanized lands impact the plant and soil microbial communities within the surrounding grasslands. We proposed the following predictions: (1) The town in pastoral area would have a negative impact on the plant and soil microbial communities of the surrounding grasslands, and this negative impact can only be detected within a specific range; (2) Urbanized lands could potentially disrupt the intricate interactions between soil microorganisms and plant communities in the surrounding grasslands. Based on the results of these predictions, we proposed recommendations aimed at achieving eco-sustainable “pastoralist relocation to towns.”

## 2 Materials and methods

### 2.1 Study sites

The study sites are situated in Xin Barag Right Banner of HulunBuir (47°36′00″N ~ 49°50′0″N, 115°31′00″E ~ 117°43′00″E), where typical temperate grassland are distributed. This region has an annual average temperature of 1.6°C, annual average precipitation of 243.9 mm, and annual average wind speed of 33 m/s. The primary soil type is Calcic Luvisols. By 2021, the urban population in the area has reached 25,206 people.

### 2.2 Sample design

**Figure 1:**
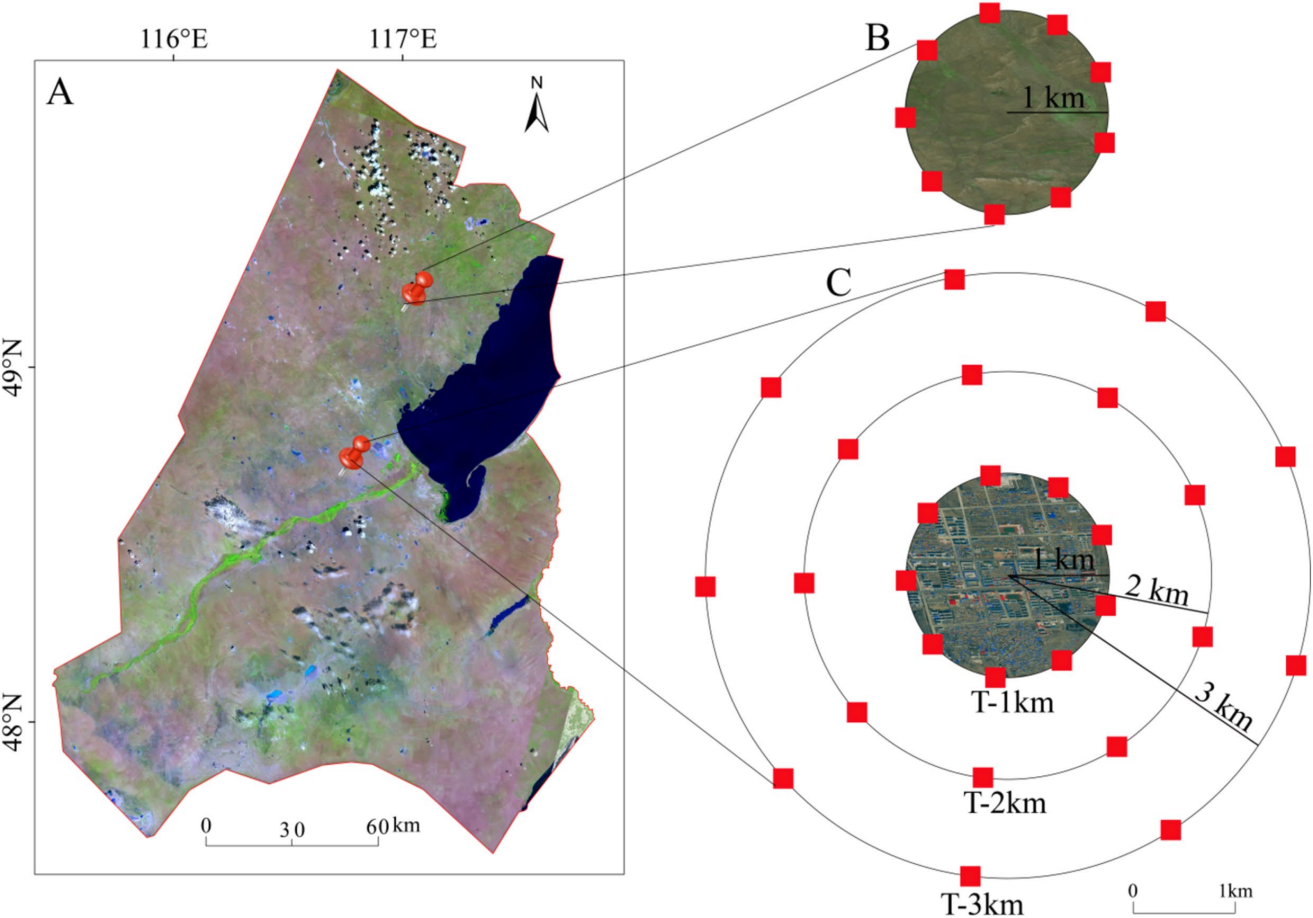
Schematic diagram of the sample design. **(A)** Xin Barag Right Banner; **(B)** control samples of the nature grassland; **(C)** experimental samples of the town. In **(B,C)** the red squares represent the 5 m × 5 m plots.

In July 2022, we selected a town sample with a radius of approximately 1 km and an area of about 4 km^2^, and a natural control sample of natural grassland with an area of 4 km^2^ located 60 km from the town (Figure 1A). The town was established in the 1980s. Because the edge between the city and the grassland is approximately 1 km from the city center, 1 km was used as a unit to detect the range of the negative edge effect. To determine the range that the negative edge effect can be detected, sample circles with radii of 1 km, 2 km, and 3 km (hereafter referred to as T-1 km, T-2 km, and T-3 km) were set up, respectively, around the town sample (Figure 1C), and a sample circle with 1 km radius (NG) was set up on the nature grassland sample (Figure 1B). On the sample circles, a 5 m × 5 m plot was set every 40° for plant community investigation and soil sample collection. Each sample circle contains 9 sample plots, with a total of 36 plots (Figures 1B,C).

### 2.3 Plant community survey

We identified the plant species and count the number of these species within each 5 m × 5 m plot. The number of plant species within each plot was used as a measure of species richness, and Shannon index was used to evaluate plant diversity. The Shannon index = $-\overset{N}{\underset{i=1}{\sum }}\left(\mathrm{Pi}*\ln\mathrm{Pi}\right)$ , in which *Pi* represents the proportion of the *i*-th species among all plant individuals in each plot.

### 2.4 Soil sampling and analysis

#### 2.4.1 Soil sampling

We collected five soil samples with a diameter of 5 cm and a depth of 20 cm from the corners and the center of each plot. The samples from one plot were mixed after passing through a 2 mm sieve and divided into three parts. The samples used for measuring soil water content (SWC) and pH were stored at 4°C, the samples used for measuring soil element content were air-dried, and the samples used for high-throughput sequencing of soil microbes were stored at −80°C.

#### 2.4.2 Soil physicochemical property analysis

The soil samples were weighed after collection and after oven dry (60°C, 24 h) respectively. The difference between the two weights is the SWC. The pH values of the soil sample suspensions (soil:water = 1:2.5) were measured by a pH meter (Sartorius PB-10, Gottingen, Germany). The total nitrogen (TN) and total organic carbon (TOC) contents were analyzed a carbon-nitrogen analyzer (Vario Max CN, Elementar, Germany). The total phosphorus (TP) content was measured by ammonium molybdate spectrophotometry. The available potassium (AK) content was measured by flame atomic absorption spectroscopy.

#### 2.4.3 Soil microbial analysis

The microbial DNA in soil samples were extracted using the Ezup Genomic DNA Extraction Kit (Sangon Biotech, Shanghai, China), and the purity and concentration of the DNA were measured using a Nanodrop 2000 spectrophotometer (Thermo Scientific, IL, Waltham, United States). Then Polymerase Chain Reaction (PCR) was used to amplify the microbial DNA. The primers used in amplifying the V4-V5 region of the bacterial 16S rRNA gene were 515F (5′-CCCCGYCAATTCMTRAGT-3′) and 909R (5′-GTGYCAGCCGGTA-3′), and used in amplifying the ITS region of fungi were ITS4 (5′-TCCTCCGCTTATTGATATGC-3′) and ITS7 (5′-GTGARTCATCGARTCTTTG-3′). The PCR reaction system containing 2 μL of DNA template, 14 μL of MIX, 12 μL of sterile water, and 1 μL of each primer. The PCR performed as the following conditions for bacteria and fungi respectively: for bacteria, initial denaturation at 94°C for 3 min, denaturation at 94°C for 40 s, annealing at 56°C for 1 min, extension at 72°C for 1 min, 33 cycles, and a final extension at 72°C for 10 min; For fungi, initial denaturation at 95°C for 5 min, denaturation at 95°C for 1 min, annealing at 53°C for 40 s, extension at 72°C for 1 min, 38 cycles, and a final extension at 72°C for 10 min. The PCR products were separated by electrophoresis on a 1% agarose gel, and recovered using the SanPrep DNA Gel Extraction Kit (Sangon Biotech, China). The samples were sequenced using the Illumina MiSeq system (Illumina, San Diego, CA, United States) to generate paired-end sequences of 2 × 250 bp. The sequence data were analyzed using the QIIME v1.9.0 platform.^1^ The paired-end sequences were assembled, matched, and taxonomically annotated using Flash 1.2.8. Chimera sequences were removed with Usearch 7.0, and sample sequences were clustered at 97% similarity using the Uclust algorithm to define operational taxonomic units (OTUs). The sequences were standardized using the website of Daisychopper.^2^ The soil microbial community alpha diversity was indicated by OTU number, Shannon and Simpson indexes. Soil bacteria and fungi functional guilds were predicted using FAPROTAX and FUNGuild.

### 2.5 Statistical analysis

One-way ANOVA with Tukey’s HSD tests were used to determine the significance of differences in the alpha diversity of plant and soil microbial communities, the abundance of dominant phyla and geuns, the abundance of functional guilds of soil microbial community, and soil physicochemical properties among different types of grasslands. Based on the Z-score of the average abundance of each plant species in T-1 km, T-2 km, T-3 km and NG, a clustering analysis on the composition of plant communities in these sample areas was performed using the Euclidean distance. OTUs with abundance >0.1% were selected, and co-occurrence networks of soil bacterial and fungal communities in different types of grassland were constructed based on the spearman correlation coefficient (|*r*| > 0.6, *p* < 0.05) (Huang et al., 2022), which was calculated using the “psych” package. The co-occurrence networks were constructed using the Fruchterman Reingold algorithm (Gephi 0.9.2). Mantel test was used to analyze the correlations among plant community, soil physicochemical properties and dominant phyla and genus of soil microorganisms using the data of T-1 km, T-2 km and NG samples. Based on the hypothesis that changes in soil properties in urban edge zones affect microbial functions and lead to plant community degradation, a prior model was constructed. Then, the prior model was modified and simplified based on the loadings of outer model, the *R*^2^ of the internal model, and the Goodness of Fit (Sanchez, 2013). The modified and simplified model includes four sets of latent variables: plant diversity, dominant species abundance, decreased soil properties, and increased soil properties, as well as three sets of manifest variable: soil bacteria phtotrophy function, wood saprotroph fungi and plant pathogen fungi functions. Finally, the impact pathways between soil properties, microbial functions, and plant communities in T-1 km and T-2 km were determined using the “plspm” package. The fitness of PLS-PM model was determined by GoF > 0.5. One-way ANOVA was conducted in SPSS 20.0. The co-network, Mantel test, and PLS-PM were performed in R4.2.2.

## 3 Results

### 3.1 Plant community composition

**Figure 2:**
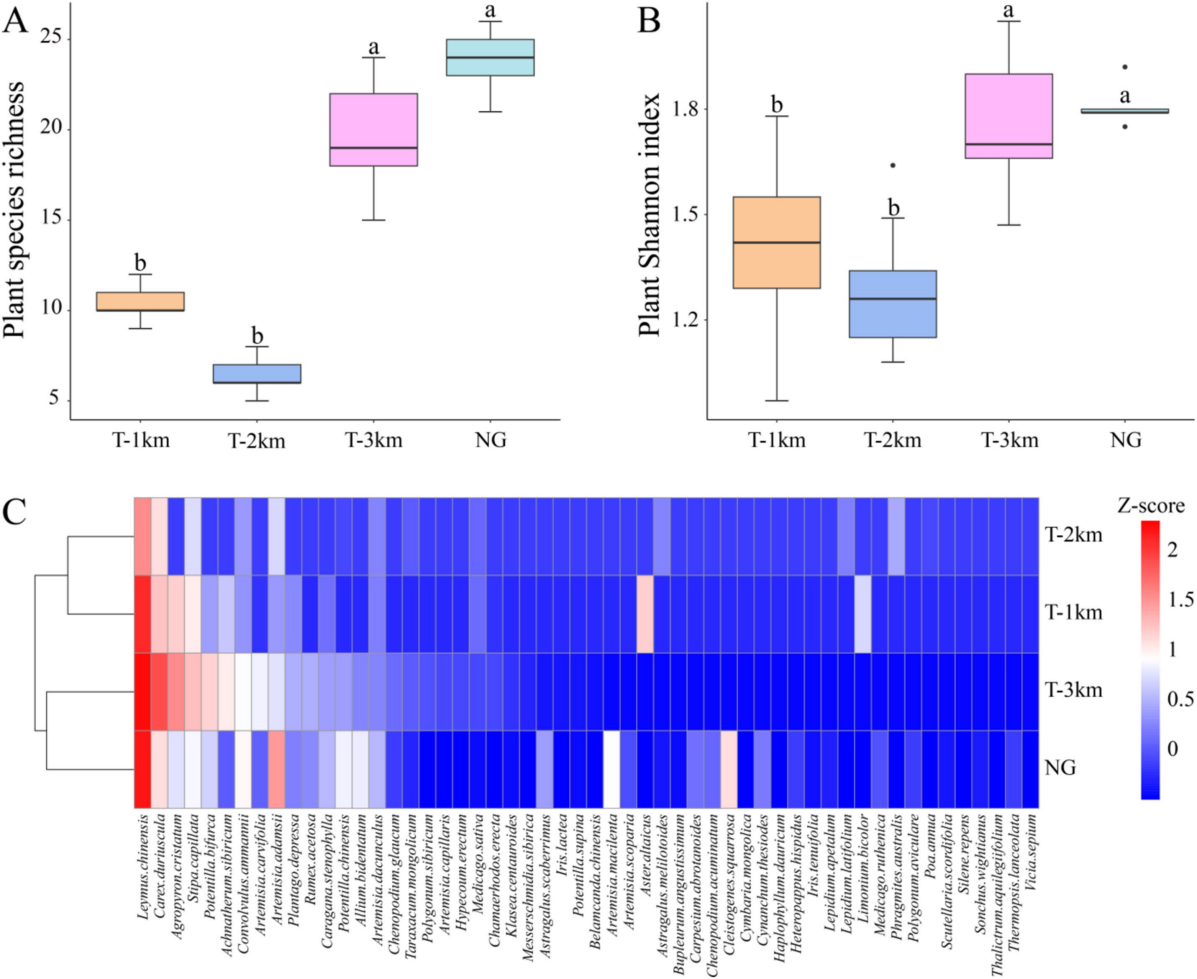
Plant species richness **(A)**, Shannon index **(B)**, and cluster analysis **(C)** of T-1 km, T-2 km, T-3 km and NG. In **(A,B)**, a, b indicated significant differences between different types of samples (One-way ANOVA, Tukey HSD test, *p* < 0.05). In **(C)** Z-score is the average abundance of each plant species.

The plant species richness and Shannon index of T-1 km and T-2 km are significantly lower than those of T-3 km and NG (One-way ANOVA, Tukey HSD test, *p* < 0.001; Figures 2A,B). *Leymus chinensis*, *Carex duriuscula*, *Stipa capillata* and *Agropyron cristatum* are the dominant plant species in T-1 km, T-2 km, T-3 km and NG samples (Figure 2C). T-1 km and T-2 km cluster together, while T-3 km and NG cluster together (Figure 2C).

### 3.2 Soil microbial community composition

**Figure 3:**
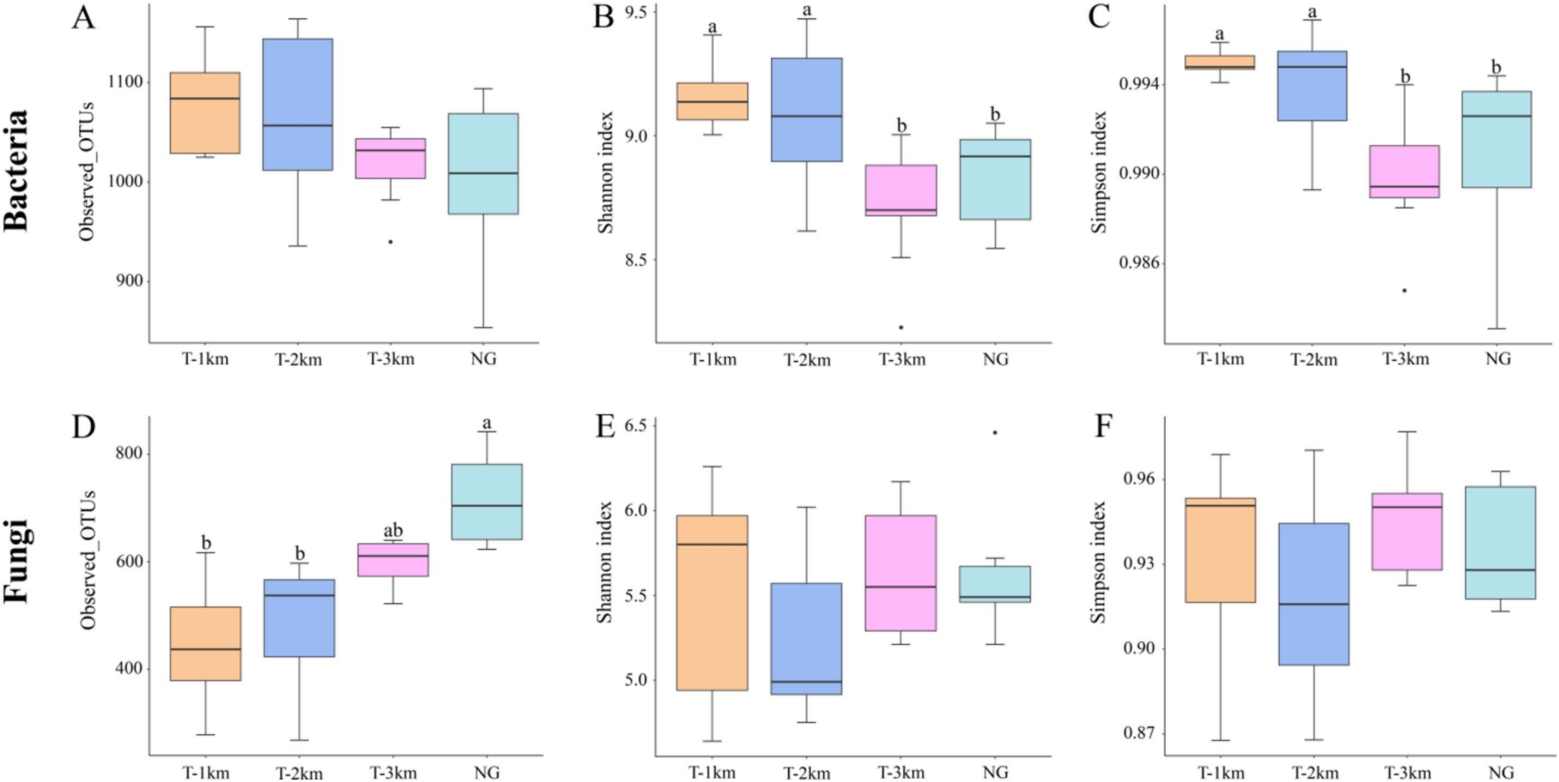
The alpha diversity of bacteria and fungi in T-1 km, T-2 km, T-3 km and NG. **(A)** Bacterial OTU number; **(B)** bacterial Shannon index; **(C)** bacterial Simpson index; **(D)** fungal OTU number; **(E)** fungal Shannon index; **(F)** fungal Simpson index. Letters a and b indicated significant differences among T-1 km, T-2 km, T-3 km and NG (One-way ANOVA, Tukey HSD test, *p* < 0.05).

**Figure 4:**
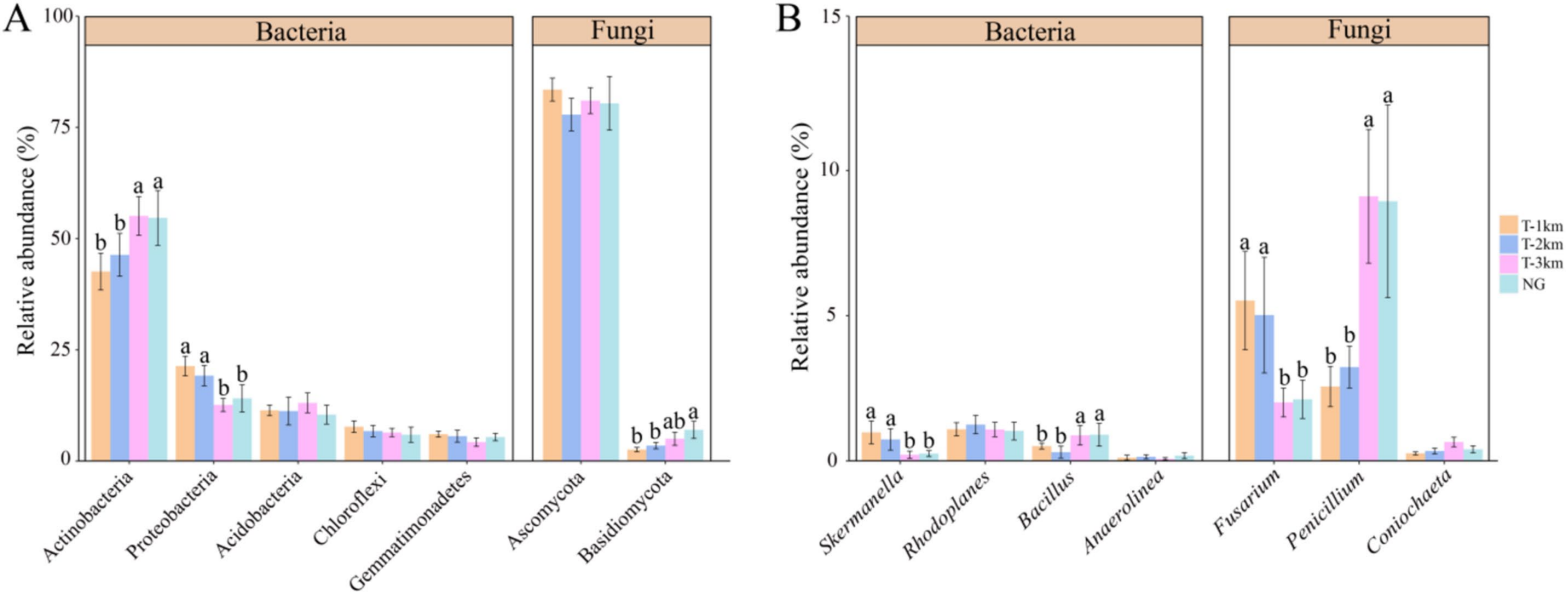
The abundance of dominant phyla **(A)** and dominant genus **(B)** of bacteria and fungi in T-1 km, T-2 km, T-3 km and NG. Abundance of dominant phyla was mean ± standard deviation. In **(A,B)**, a, b indicated significant differences between different types of samples (One-way ANOVA, Tukey HSD test, *p* < 0.05).

**Figure 5:**
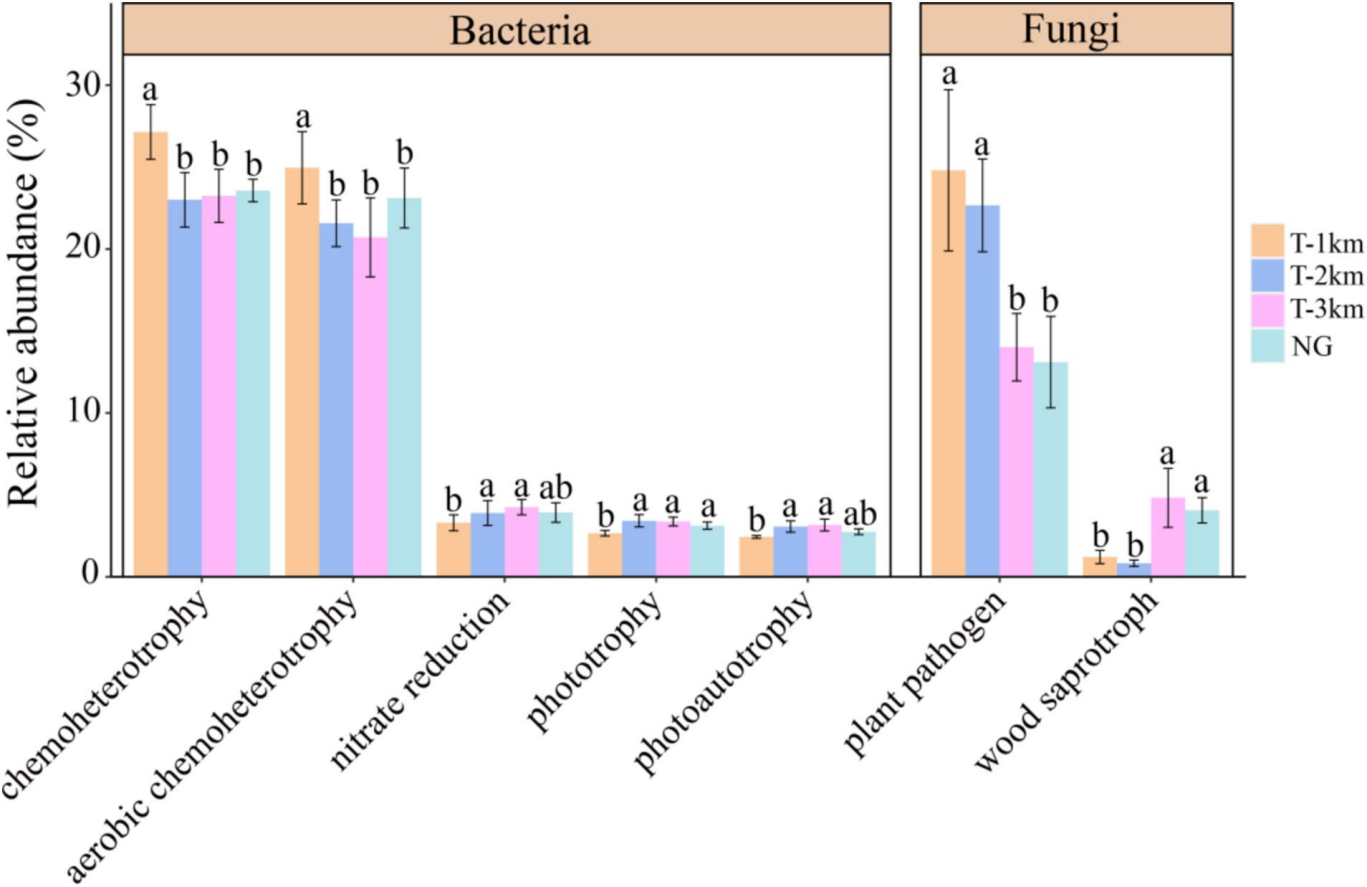
The bacterial and fungal functional guilds with significant difference in abundance among groups (T-1 km, T-2 km, T-3 km and NG). Abundance of functional guilds was mean ± standard deviation; a, b indicated significant differences between different types of samples (One-way ANOVA, Tukey HSD test, *p* < 0.05).

**Figure 6:**
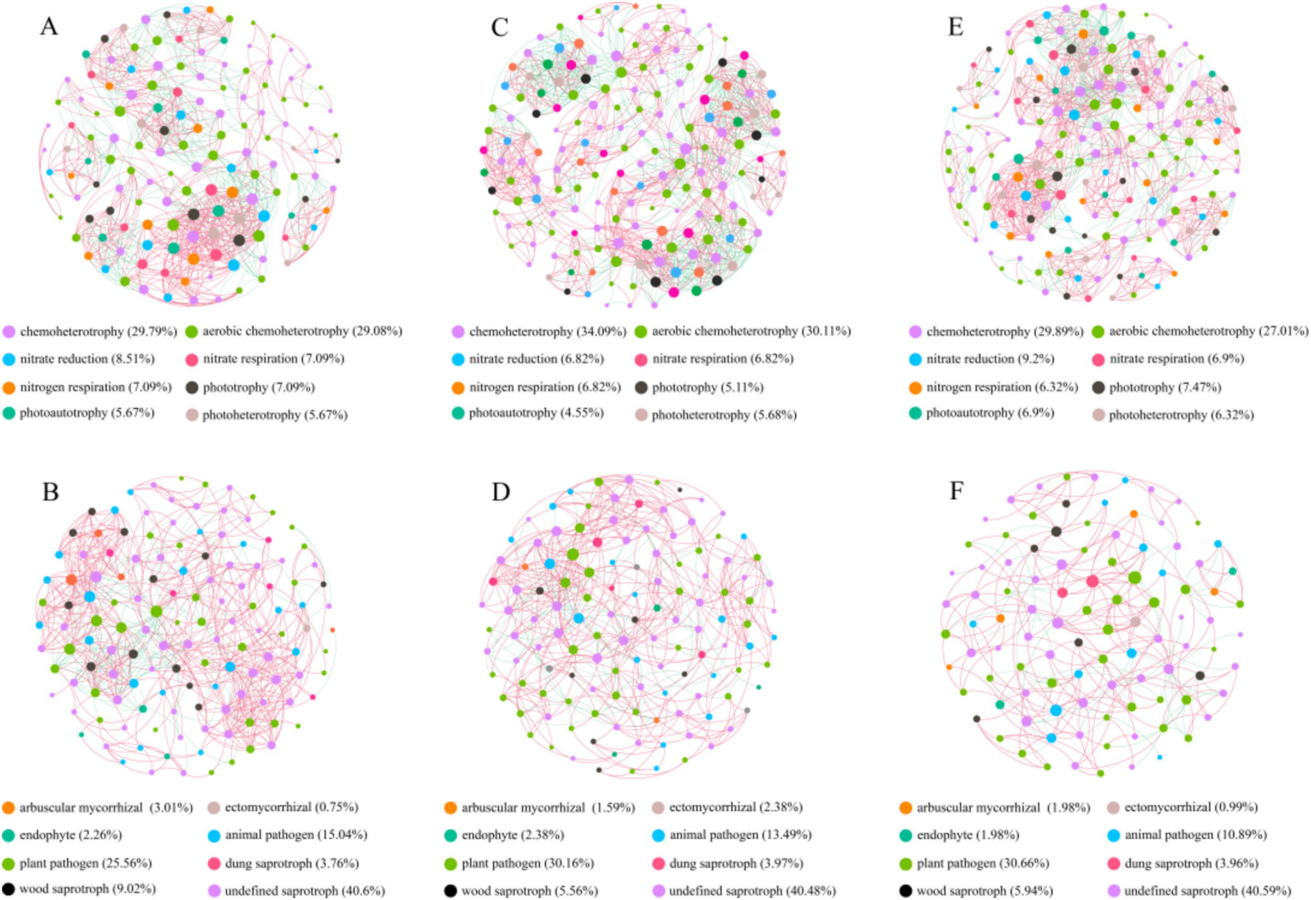
The co-occurrence networks of bacteria and fungi in NG **(A,B)**, T-1 km **(C,D)** and T-2 km **(E,F)**. Red lines and green lines indicate positive and negative correlations, respectively. Points with different colors represented OTUs at different functional guilds.

Comparing to NG, the bacteria Shannon and Simpson index in T-1 km and T-2 km significantly increase (One-way ANOVA, Tukey HSD test, *p* < 0.05) and the fungal OTU numbers in T-1 km and T-2 km significantly decrease (One-way ANOVA, Tukey HSD test, *p* < 0.05) (Figure 3). In T-1 km and T-2 km samples, the abundance of Actinobacteria and Basidiomycota phylums, *Bacillus* and *Penicillium* genus significantly decrease, while the abundance of Proteobacteria phylum, *Skermanella* and *Fusarium* genus significantly increase (One-way ANOVA, Tukey HSD test, *p* < 0.05, Figure 4). Comparing to NG, the abundance of chemoheterotrophy and aerobic chemoheterotrophy bacteria significantly increase, while the abundance of phototrophy, nitrate reduction and photoautotrophy bacteria significantly decrease in T-1 km (One-way ANOVA, Tukey HSD test, *p* < 0.05); the abundance of plant pathogen fungi significantly increased, while the abundance of wood saprotroph fungi significantly decreased in T-1 km and T-2 km (One-way ANOVA, Tukey HSD test, *p* < 0.05) (Figure 5). The bacterial co-ocurrence networks in T-1 km has higher node number, edge number, average path length; the fungal co-ocurrence networks in T-1 km and T-2 km have lower node number, edge number, average degrees and average clustering coefficient, but higher average path lengths and positive correlation percentage (Figure 6; Table 1).

**Table 1:** The co-occurrence network topological characteristics of soil bacterial and fungal communities in NG, T-1 km andT-2 km.

Network properties	NG	T-1 km		T-2 km		
	Bacteria	Fungi	Bacteria	Fungi	Bacteria	Fungi
Node number	141	133	176	126	174	101
Edge number	893	638	1,214	486	1,056	264
Average degree	12.67	9.59	13.80	7.71	12.14	5.23
Average path length	3.67	3.01	3.93	3.38	4.61	4.08
Average clustering coefficient	0.76	0.55	0.74	0.49	0.78	0.47
Positive correlation (%)	61.48	77.59	56.34	81.89	66.48	79.17
Negative correlation (%)	38.52	22.41	43.66	18.11	33.52	20.83

### 3.3 Soil physicochemical properties

The SWC and TOC content of T-1 km and T-2 km samples were significantly lower than that of T-3 km and NG, while the pH and TP content were significantly higher than that of T-3 km and NG (One-way ANOVA, Tukey HSD test, *p* < 0.05; Table 2).

**Table 2:** Soil physicochemical properties in T-1 km, T-2 km, T-3 km and NG.

	T-1 km	T-2 km	T-3 km	NG
SWC (%)	4.01 ± 0.12^b^	4.07 ± 0.10^b^	4.87 ± 0.78^a^	5.06 ± 0.68^a^
pH	8.68 ± 0.19^a^	8.61 ± 0.25^a^	7.84 ± 0.46^b^	7.75 ± 0.34^b^
TOC (g/kg)	16.44 ± 1.37^b^	18.46 ± 0.81^b^	22.60 ± 0.77^a^	23.23 ± 4.52^a^
TP (g/kg)	0.79 ± 0.07^a^	0.74 ± 0.04^a^	0.53 ± 0.06^b^	0.58 ± 0.09^b^
TN (g/kg)	2.23 ± 0.28^a^	1.93 ± 0.26^a^	2.00 ± 0.33^a^	2.30 ± 0.33^a^
K (g/kg)	0.86 ± 0.06^a^	1.04 ± 0.31^a^	0.94 ± 0.15^a^	0.95 ± 0.08^a^

Letters a, b indicated significant differences between different samples (One-way ANOVA, Tukey HSD test, *p* < 0.05).

### 3.4 Correlations between plant community, soil properties and soil microbial community

**Figure 7:**
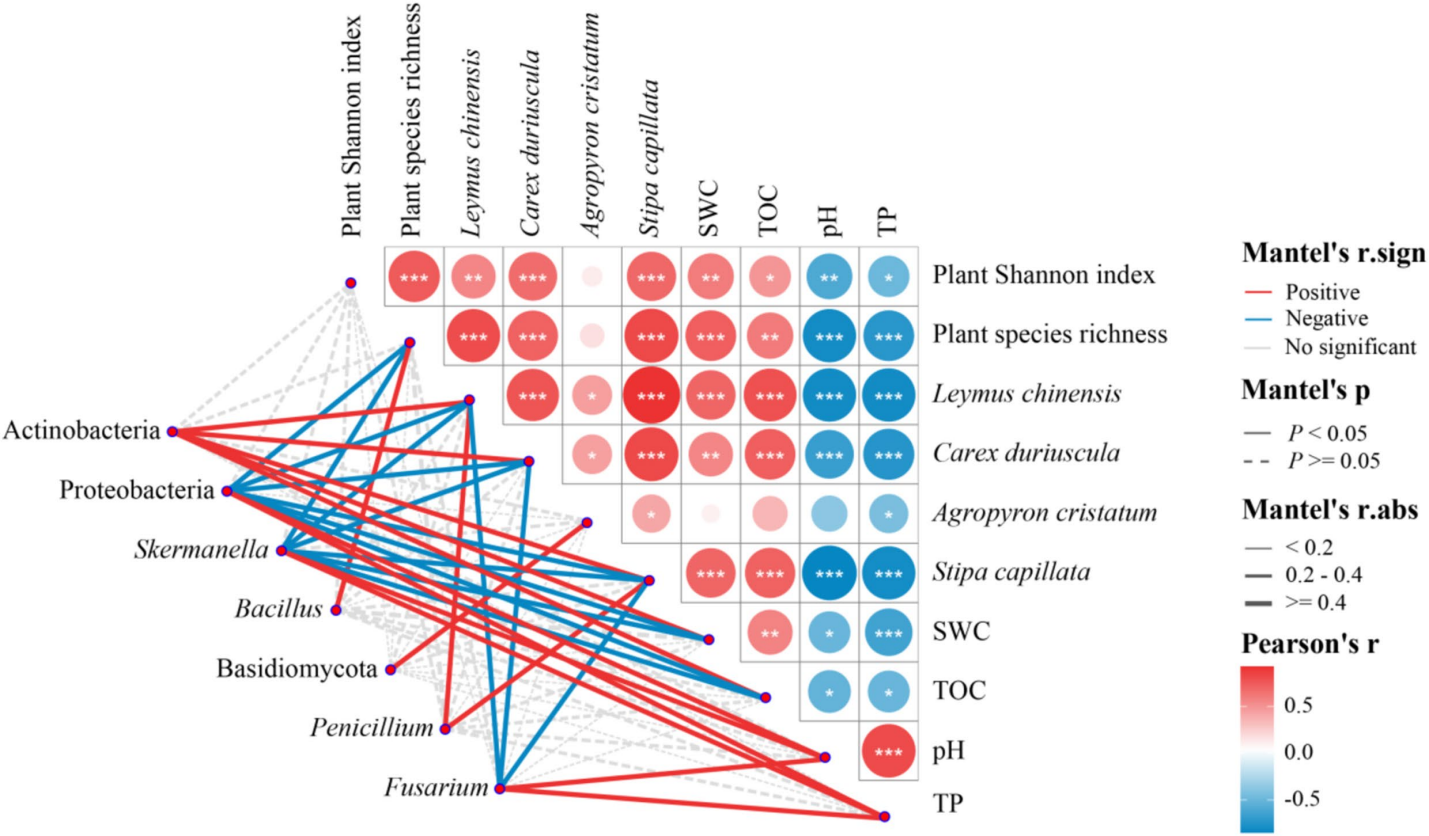
Mantel test on the correlations among plant diversity, dominant plant species abundance, soil properties, and soil bacteria and fungi phyla in T-1 km, T-2 km and NG samples.

Plant species richness, plant Shannon index, the abundance of *Leymus chinensis*, *Carex duriuscula* and *Stipa capillata* were positively correlated with SWC and TOC and negatively correlated with pH and TP (Figure 7). Actinobacteria was positively correlated with SWC, TOC, and the abundance of *Leymus chinensis*, *Carex duriuscula*, *Stipa capillata*; *Bacillus* was positively correlated with plant species richness; Proteobacteria and *Skermanella* were positively correlated with pH and TP and negatively correlated with SWC, TOC, plant species richness, and the abundance of *Leymus chinensis*, *Carex duriuscula*, *Stipa capillata*. Basidiomycota was positively correlated with the abundance of *Agropyron cristatum*, *Penicillium* was positively correlated with the abundance of *Leymus chinensis* and *Stipa capillata*; *Fusarium* was positively correlated with pH and TP, and negatively correlated with the abundance of *Leymus chinensis*, *Carex duriuscula*, *Stipa capillata* (Figure 7).

### 3.5 Path model for the plant–soil interactions in grassland surrounding the town

**Figure 8:**
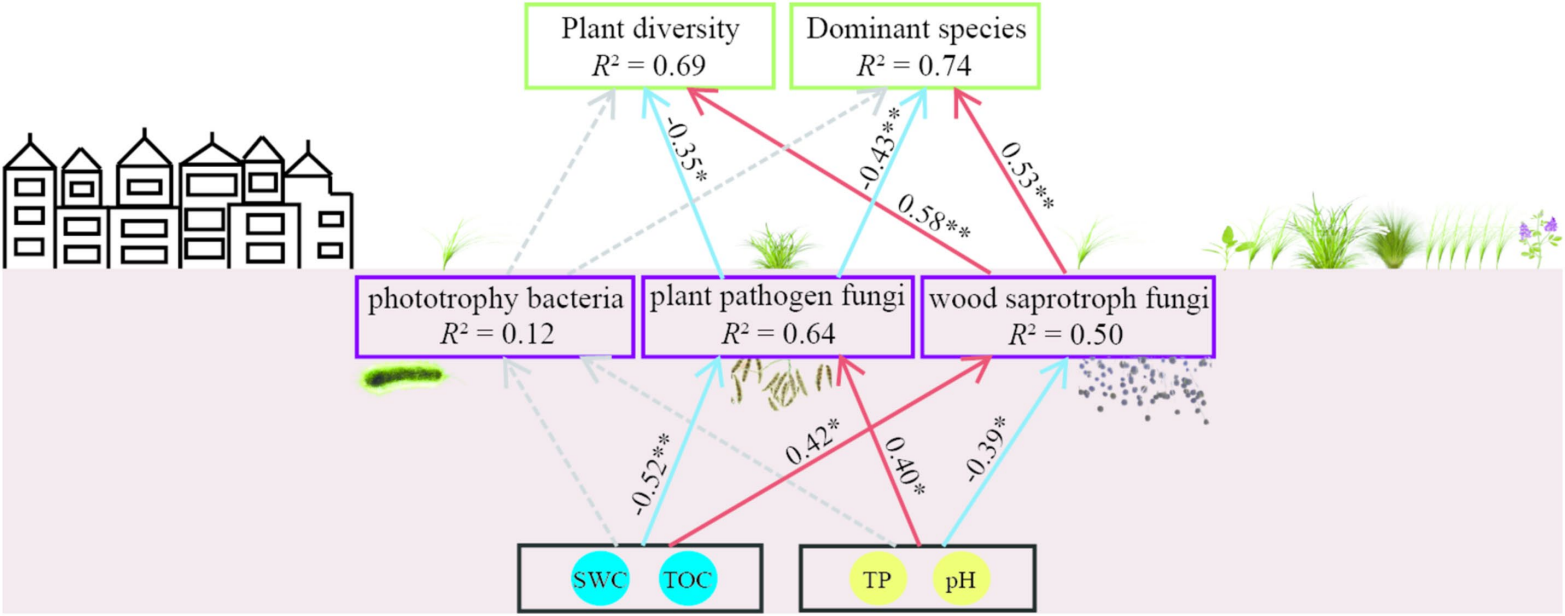
The partial least squares path model of plant diversity, dominant species, soil microbial function, and soil properties in T-1 km and T-2 km. Red and blue arrows represent positive and negative effects (*p* < 0.05), respectively. The dashed line represents a non-significant relationship (*p* > 0.05).

A partial least squares path model (GoF = 0.69) was constructed using seven variables in this study: plant diversity (plant species richness and plant Shannon index), dominant species abundance (*Leymus chinensis*, *Carex duriuscula*, and *Stipa capillata*), decreased soil properties (SWC, TOC) and increased soil properties (pH, TP), soil bacteria phtotrophy function, wood saprotroph fungi and plant pathogen fungi functions. In the model, SWC and TOC had negative and positive direct effects on plant pathogen and wood saprotroph, respectively; pH and TP had positive and negative direct effects on plant pathogen and wood saprotroph respectively; plant pathogen had a negative direct effect on plant diversity and dominant species, while wood saprotroph had a positive direct effect on plant diversity and dominant species (Figure 8).

## 4 Discussion

The significant decreases in plant diversity, soil fungi abundance, soil water content (SWC), and total organic carbon (TOC) observed in T-1 km suggest a negative edge effect induced by the town. This may be related to changes in building density and population concentration in urban areas (Gao D. D. et al., 2023; Li L. et al., 2024; Yang and Wu, 2024). The interference of urbanization and human activities may lead to the deterioration of soil properties around cities, reduce the diversity of plant and soil microbial communities, and exacerbate grassland degradation (Gao D. D. et al., 2023). Furthermore, the similarity in plant and fungi communities, along with soil properties, between T-2 km and T-1 km zones indicates that the adverse edge effect on the surrounding grasslands can be detected within a 1 km range from the perimeter of the town. Moreover, according to the mechanism of negative edge effect, degraded areas may continue to expand to the surrounding areas.

The significant decline in fungal α diversity and the abundance of soil microorganisms crucial for nutrient cycling, coupled with a marked increase in bacterial α diversity and microorganisms which causing plant diseases, was observed in the T-1 km and T-2 km zones. Bacterial diversity is more sensitive to changes in soil phosphorus and pH, while fungal diversity is more sensitive to changes in soil organic matter and moisture content (Hermans et al., 2020; Philippot et al., 2024). The increased soil phosphorus can provide more nutrients for bacteria, and increased soil pH can promote phosphorus absorption by bacteria and promote the proliferation of alkaline bacteria (such as Proteobacteria) (Liu et al., 2014; Tian et al., 2024). Therefore, increase in soil phosphorus (TP) content and pH value in the T-1 km and T-2 km may explain the increase in bacterial alpha diversity. Soil organic carbon is the most important carbon source for the growth and reproduction of soil fungi, and soil moisture is the foundation for maintaining fungal metabolism (Che et al., 2018; Che et al., 2019). The decrease in SWC and TOC in T-1 km and T-2 km may directly affect the survival and reproduction of fungi, thereby reducing fungal α diversity. Notably, Actinobacteria play a pivotal role in soil carbon cycling (Francioli et al., 2016; Meng et al., 2023), while Basidiomycota facilitate the decomposition of recalcitrant organic matter like lignin in soil (Voriskova and Baldrian, 2013; Wu et al., 2021). Additionally, *Bacillus* and *Penicillium* are known for decomposing cellulose and lignin from plant residues (Li R. C. et al., 2021). Therefore, the reduction of these bacteria and fungi in the T-1 km and T-2 km zones may decrease the efficiency of soil nutrient cycling. Conversely, *Fusarium* can infect plant roots and stems, leading to wilting and plant death (Stępień, 2023), thus its increase in the T-1 km and T-2 km zones may lead to the degradation of plant communities. Furthermore, microorganisms indicative of urbanization effects, such as human gut-associated Proteobacteria and drought-tolerant *Skermanella*, significantly increased in the T-1 km and T-2 km zones (Li et al., 2018; Wang et al., 2018; Yadav and Seob, 2016). The functional guild analysis corroborates the findings from phylum and genus composition analyses, revealing a decline in phototrophy, nitrate reduction bacteria, and wood saprotroph fungi. Phototrophy and nitrate reduction bacteria are important bacteria involved in carbon fixation and nitrate conversion (Gardner et al., 2006; Hamard et al., 2021). Wood saprotroph fungi can convert lignin and cellulose in plant residues into nutrients that plants can utilize (Cline et al., 2017; Wang et al., 2020). The decrease in the abundance of phototrophy, nitrate reduction bacteria and wood saprotroph fungi may hinder the decomposition process, thus reduce the nutrient availability in the soil and negatively impact plant diversity. Conversely, stress-resistant chemoheterotrophic and aerobic chemoheterotrophic bacteria increased within a 2 km radius from the town center. The chemoheterotrophic and aerobic chemoheterotrophic bacteria can obtain energy by oxidizing inorganic chemicals (such as sulfur, iron, or ammonia) or degrading refractory organic matter in nutrient-deficient soil (Rolando et al., 2022; Labouyrie et al., 2023), which allows them to maintain high abundance even in poor soil environment. Therefore, the increase in chemoheterotrophic and aerobic chemoheterotrophic bacteria indicate the significant soil degradation in T-1 km. In addition, plant pathogenic fungi significantly increased at T-1 km and T-2 km. The increase in their abundance may lead to a decline in plant diversity and productivity by increasing the probability of plant diseases (Fisher et al., 2012). These alterations in soil microbial community diversity, composition and function in T-1 km and T-2 km suggest that the negative edge effects induced by town extend beyond the immediate town boundaries, particularly impacting fungal composition and function across a broader area.

The microbial co-occurrence network serves as a reflection of the adaptability of microbial communities to environmental stress (de Vries et al., 2018; Zheng et al., 2021; Chen et al., 2022). Generally, bacteria possess higher reproductive capacity and resilience to stress (Zhang et al., 2020). They can adjust the proportions of key groups and the strength of interactions within the network in response to external disturbances, thereby enhancing the stability and complexity of the network (Xun et al., 2018; Zhang et al., 2020; Scholier et al., 2023). Conversely, fungi exhibit closer interactions between functional guilds and are more vulnerable and sensitive to negative disturbances. Therefore, a decrease in the complexity of fungal co-occurrence networks indicates grassland degradation (Wu et al., 2021; Wang et al., 2022a). Our findings that the complexity of co-occurrence networks increased for bacteria but decreased for fungi in the T-1 km and T-2 km areas suggest that the soil microbial community in the 1 km surrounding the town suffered negative environmental stress and underwent degradation.

Urbanized lands can alter the soil properties in surrounding natural ecosystems, thus negatively disrupting the interaction between plants and soil (Malmivaara-Lämsä et al., 2008; Tatsumi et al., 2023). The decrease in total organic carbon (TOC) and soil water content (SWC) may reduce the metabolic rate of wood saprotrophic fungi and the disease resistance ability of plants (Manzoni et al., 2012; Davinic et al., 2013; Choudhary and Senthil-Kumar, 2024). Conversely, the increase in pH and total phosphorus (TP) can decrease the activity of wood saprotrophic fungi and promote the colonization of plant pathogens (Fierer, 2017; Li P. F. et al., 2021; Geng et al., 2023). The results of the Mantel test and path model in this study collectively indicate that the plant–soil interaction in the area within 1 km surrounding the town suffered negative disturbance. The plant community diversity and dominant species abundance decreased with the decrease in SWC and TOC. This degradation of the plant community may reduce soil nutrient input, thereby decreasing the abundance of microorganisms involved in soil nutrient cycling, such as Actinobacteria, *Bacillus*, *Penicillium*, and Basidiomycota (Li H. Y. et al., 2021; Wu et al., 2021). The decrease in wood saprotrophic fungi can lead to a reduction in carbon cycling rate (Cline et al., 2017; Wang et al., 2020), further decreasing TOC. The increase in soil TP and pH can promote the rapid propagation of plant pathogen fungi, such as *Fusarium* (Elgharably and Marschner, 2011; Yang et al., 2022b; Zhang et al., 2023). The increase in plant pathogen fungi can reduce photosynthetic efficiency (Fisher et al., 2012), leading to plant biomass loss (Allan et al., 2010; Seabloom et al., 2017; Jia et al., 2020). The negative correlation between plant diversity and pathogen fungi abundance has been verified in many grasslands (Li et al., 2016; Halliday et al., 2020; Zhang et al., 2024). The results of the path model showed that the decrease in SWC and TOC and the increase in pH and TP in the area within 1 km surrounding the town can lead to a decrease in wood saprotrophic fungi and an increase in plant pathogen fungi, ultimately resulting in a decrease in plant diversity and dominant species in these areas. These results support our prediction that towns degrade the surrounding grasslands by deteriorating plant–soil interactions.

Although many studies have evaluated the negative edge effects of urbanized lands on surrounding natural ecosystems, most of these studies have been conducted in urban-forest edges (Smith and Smith, 2010; Vallet et al., 2010; Veselkin et al., 2018; Garvey et al., 2022; Tatsumi et al., 2023). Whether and how urbanization-induced negative effects exist in grassland ecosystems remains unclear. Compared to forest ecosystems, grasslands are fragile ecosystems due to poor soil and low rainfall (Gao B. et al., 2023; Liu et al., 2024). Thus, the negative edge effect on plant–soil interactions caused by urbanization is expected to be more significant in grassland ecosystems. Our results show that the negative edge effect on plant–soil interactions can be detected within a range of 1 km from the perimeter of the town, while available studies in forests have shown that the range of the negative edge effect is only 10-40 m (Smith and Smith, 2010; Vallet et al., 2010; Veselkin et al., 2018; Garvey et al., 2022). Our results indicate that the fragile grassland ecosystems are more susceptible to the negative edge effects caused by urbanization than forest ecosystems. For this reason, the towns in pastoral regions should be planned based on the range of the negative edge effect.

Our results indicate that urban construction can lead to a broader surrounding grassland degradation by negative edge effect. The degradation of vegetation adjacent to the town may further reduce nutrient inputs and disrupt water cycle in there adjacent grassland ecosystems, then consequently deteriorate the plant–soil microbiome interactions, and degrade more vegetation. Our results provide valuable informations for planning towns in pastoral regions. Firstly, town construction not only transforms grassland into urbanized land but also leads to grassland degradation expansion in the surrounding area. Secondly, the negative effect on the grassland can be detected within a 1 km radius surrounding the town edge. According to this, the area of the town constructed in grassland ecosystem should be restricted as small as possible in order to decrease the disturbance on the surrounding grassland ecosystem. It is necessary to consider the negative impacts of edge effects on the grassland ecosystem when constructing towns to achieve sustainable pastoralist relocation. Our study area is located in temperate meadow grassland, where the annual precipitation and soil nutrient content (especially total organic matter and total nitrogen) are higher than those in typical grassland and desert grassland (Lu et al., 2024). Therefore, it can be predicted that urbanization in typical grassland and desert grassland may lead to more extensive grassland degradation around urban due to negative edge effects.

## 5 Conclusion

Within a 1 km radius surrounding towns, there was a significant decrease in soil total organic carbon and soil water content, and a significant increase in soil total phosphorus and pH. These changes in soil properties led to a decrease in the abundance of fungi related to nutrient cycling and an increase in plant pathogens, which resulted in a decrease in plant diversity and abundance of dominant plant species, ultimately leading to surrounding grassland degradation. The urbanization-induced negative edge effect is more significant in grasslands than in forests. The negative impact of edge effects should be considered when constructing towns on grasslands.

## Data Availability

Microbial raw sequence data that support the findings of this study are openly available in NCBI at Sequence Read Archive (SRA), reference number PRJNA1171968 (bacteria) and PRJNA1171979 (fungi).

## Footnotes

^1^ http://qiime.org/tutorials/tutorial.html

^2^ http://www.genomics.ceh.ac.uk/GeneSwytch/Tools.html
